# Prevalence and distribution of cervical high-risk human papillomavirus and cytological abnormalities in women living with HIV in Denmark – the SHADE

**DOI:** 10.1186/s12885-016-2881-1

**Published:** 2016-11-08

**Authors:** Kristina Thorsteinsson, Merete Storgaard, Terese L. Katzenstein, Steen Ladelund, Frederikke Falkencrone Rønsholt, Isik Somuncu Johansen, Gitte Pedersen, Lailoma Hashemi, Lars Nørregård Nielsen, Lisbeth Nilas, Niels Obel, Jesper Bonde, Anne-Mette Lebech

**Affiliations:** 1 Department of Infectious Diseases, Hvidovre, Copenhagen University Hospital, Copenhagen, Denmark; 2 Department of Infectious Diseases, Skejby, Aarhus University Hospital, Aarhus, Denmark; 3 Department of Infectious Diseases, Copenhagen University Hospital, Rigshospitalet, Copenhagen, Denmark; 4 Institute of Clinical Medicine, University of Copenhagen, Copenhagen, Denmark; 5 Clinical Research Center, Hvidovre, Copenhagen University Hospital, Copenhagen, Denmark; 6 Department of Infectious Diseases, Odense University Hospital, Odense, Denmark; 7 Department of Infectious Diseases, Aalborg University Hospital, Aalborg, Denmark; 8 Department of Obstetrics and Gynaecology, Aalborg University Hospital, Aalborg, Denmark; 9 Department of Infectious Diseases, Nordsjællands Hospital, Hillerød, Denmark; 10 Department of Obstetrics and Gynaecology, Hvidovre, Copenhagen University Hospital, Copenhagen, Denmark; 11 Molecular Pathology Laboratory, Department of Pathology, Hvidovre, Copenhagen University Hospital, Copenhagen, Denmark; 12 Department of Infectious Diseases, Hvidovre Hospital, Kettegaards Allé 30, 2650 Hvidovre, Denmark

**Keywords:** Women living with HIV, High-risk human papillomavirus, Cervical cytological abnormalities, HPV vaccine, Highly active antiretroviral therapy

## Abstract

**Background:**

Women living with HIV (WLWH) are at increased risk of persistent human papillomavirus (HPV) infection, cervical dysplasia and cervical cancer compared with women from the general population (WGP). We assessed the prevalence and distribution of cervical high-risk (hr) HPV infection and cytological abnormalities in WLWH compared with WGP in Denmark. Predictors of HPV and cytological abnormalities were estimated in WLWH.

**Methods:**

WLWH consecutively enrolled in the **S**tudy on **H**IV, cervical **A**bnormalities and infections in women in **De**nmark (SHADE) in 2011 and were examined for cervical HPV and cytological abnormalities.

WLWH were matched on age and prior cytological findings with WGP from an earlier study. HIV demographics were retrieved from the nationwide Danish HIV Cohort Study. Logistic regression was used to estimate predictors of hrHPV and cytological abnormalities.

**Results:**

Of 334 included WLWH 26.4 % were positive for hrHPV as opposed to 16.6 % WGP (*p <* 0.0001). WLWH had a higher number of multiple infections (>1 h genotype present) (38.5 % versus 25.7 %, *p =* 0.030). Hr genotypes in descending order of frequency were HPV58 (7.1 %), 52 (5.4 %), and 16 (4.8 %) in WLWH versus HPV16 (4.1 %), 52 (2.8 %) and 58 (2.4 %) in WGP. Predictors of hrHPV in WLWH were short duration of HAART (adjusted OR per year 0.90 (95 % CI 0.84-0.96)), AIDS prior to inclusion (adjusted OR 3.61 (95 % CI 1.75-7.46)), ≥5 lifetime sexual partners (adjusted OR 2.20 (95 % CI 1.08-4.49)), sexual debut <16 years of age (adjusted OR 2.05 (95 % CI 1.03-4.10)) and CD4 < 350 cells/μL (adjusted OR 2.53 (95 % CI 1.20-5.40)). Cytological abnormalities were prevalent in 10.4 % vs. 5.2 % (*p* = 0.0003) of WLWH and WGP. In WLWH with hrHPV, short duration of HAART predicted cervical dysplasia (adjusted OR per year 0.83 (95 % CI 0.71-0.97)).

**Conclusions:**

WLWH presented with more cervical hrHPV infections and cytological abnormalities, and a different distribution of hrHPV genotypes compared with WGP. Cervical hrHPV and cytological abnormalities were predicted by short duration of HAART.

**Electronic supplementary material:**

The online version of this article (doi:10.1186/s12885-016-2881-1) contains supplementary material, which is available to authorized users.

## Background

Human papillomavirus (HPV) is the most common sexually transmitted disease (STD) [1] with a lifetime prevalence of up to 80 % [2]. Essentially, all cases of cervical cancer (CC) are associated with high-risk HPV (hrHPV) [3] and worldwide approximately half a million women develop CC each year [4]. Women living with HIV (WLWH) are at increased risk of persistent HPV infection, cervical dysplasia and CC compared with women from the general population (WGP) [5–9].

Comprehensive data on the prevalence and distribution of HPV genotypes are informative in the planning of HPV/CC screening tools and with the rollout of HPV vaccines [10]. The hrHPV genotype distribution in Denmark [11, 12] and worldwide is well described in WGP and globally HPV16, 18, 52, 31, and 58 predominate [10, 13, 14]. Most studies suggest a different HPV genotype distribution in WLWH with increased frequency of non-16/18 HPV genotypes, which are not targeted by the 2-and 4-valent HPV vaccines [8, 15, 16].

Though, immune reconstitution induced by highly active antiretroviral therapy (HAART) might decrease the prevalence of HPV and cervical dysplasia, the effect of improved immunosurveillance remains controversial [6, 7]. The increased longevity gained from HAART [17] may increase risk of exposure to HPV and provide the time required for progression to cancer.

Compared with other Western countries WGP in Denmark have high incidences of HPV and CC [11, 18, 19]. However, the prevalence and distribution of cervical HPV and dysplasia are unknown in WLWH in Denmark. Denmark offers a unique setting for studies on HPV and cervical dysplasia because of the well described HPV genotype distribution in WGP in Denmark [11, 19] and the possibility for linkage to nationwide registries on HIV and cytology results.

The aim of the present study was to assess the prevalence and distribution of cervical hrHPV and cytological abnormalities in WLWH in Denmark. Further, we aimed at identifying predictors of hrHPV and cytological abnormalities in WLWH in a setting with free access to CC screening, healthcare and HAART.

## Methods

### Setting

Denmark has a population of 5.6 million [20] and an estimated HIV prevalence among adults of 0.1 % [21] - one-fourth of these being women [22]. Medical care, including HAART, is tax-paid and provided free-of-charge to all people living with HIV (PLHIV). Treatment of HIV is restricted to eight specialized centres, of which six (treating 97 % of Danish PLHIV) participated in the **S**tudy on **H**IV, cervical **A**bnormalities and infections in women in **De**nmark (SHADE) [23] (see below).

### Cervical screening in Denmark

During the study period, The Danish National Board of Health recommended that women aged 23–49 years received cervical cytological testing every three years and women aged 50–65 years every five years [24]. In HIV guidelines cervical cytology is recommended twice the first year after HIV diagnosis and annually thereafter [25].

### The SHADE cohort

The SHADE cohort is a multicentre, prospective, observational cohort study of WLWH in Denmark. Study procedures have been described previously [23]. In brief, study participants were consecutively enrolled during their outpatient visits from 1 February 2011 to 1 February 2012. Inclusion criteria were HIV-1 infection and ≥18 years of age. Exclusion criteria were prior hysterectomy, pregnancy, alcohol and/or drug abuse impeding adherence to the protocol.

### Interview survey

At entry, an interview including tobacco use, age at sexual debut, lifetime sexual partners, prior condyloma, HPV vaccination status, and contraceptive use etc. was performed. The EpiData Entry program was used for double manual data entry [26].

### Registries

#### Civil Registration System (CRS)

The CRS is a national registry of all Danish residents [27]. A 10-digit personal identification number (PIN) is assigned to each individual at birth or immigration. The PIN was used to link the SHADE cohort, the Danish HIV Cohort Study (DHCS) and the The Danish Pathology Data Bank (DPDB).

#### Danish HIV cohort study

The DHCS is a prospective, observational, nationwide, multicentre cohort study of all PLHIV seen at the Danish HIV clinics since 1 January 1995. The cohort has been described in detail elsewhere [22].

#### The Danish Pathology Data Bank (DPDB)

The DPDB contains nationwide records of all pathology specimens [28]. Cytology samples prior to inclusion were retrieved to assess screening history and cytology results using the Systemized Nomenclature of Medicine (SNOMED) code of cervix uteri: T8x2*, T8x3* and T83*.

#### Data from the general population (the Horizon Study)

Anonymised data on HPV (from the CLART assay) and cytology results from WGP included in the Danish Horizon Study, Copenhagen, Denmark, were retrieved from the authors [11].

### Cytology

Cytological evaluation of SurePath samples was undertaken at Department of Pathology, Hvidovre, Copenhagen University Hospital (HVH). The outcomes were reported using the Bethesda 2001 system [29] and classified as normal, atypical cells of undetermined significance (ASCUS), low-grade squamous intraepithelial lesions (LSIL), or high-grade squamous intraepithelial lesions (HSIL) including atypical squamous cells - cannot exclude HSIL (ASC-H), atypical glandular cells (AGC) and adenocarcinoma in situ (AIS), and finally squamous cell-and adenocarcinoma.

### HPV DNA testing

Cervical samples were examined by the CLART HPV2 assay (Genomica, Madrid, Spain) at Department of Pathology, HVH. PCR amplification of genotype specific HPV L1 fragments from 35 individual HPV genotypes was performed including 13 hrHPV: HPV16, 18, 31, 33, 35, 39, 45, 51, 52, 56, 58, 59, and 68 [11]. Samples with invalid outcomes were retested. The second result was considered definitive [11].

### Statistical analysis

Continuous variables were summarized as median and interquartile ranges (IQR) or mean and ranges and compared using the Wilcoxon rank sum test. Categorical variables were reported as counts and percentages and compared using the chi-square test or Fisher’s exact test.

For comparison of HPV status, genotype distribution and cytology results study participants were matched 1:5 to WGP on prior screening history in accordance with the algorithm used in the Horizon study [11] and age with a tolerance of 2 years (choosing the 5 WGP numerically closest to the participant’s age).

Univariate and multiple logistic regression analyses were used for identifying predictors of hrHPV expressed as odds ratios (OR) and 95 % confidence intervals (CI). Nine candidate predictor variables were chosen a priori due to current knowledge on risk factors of HPV [6, 30, 31]; age (18–29, 30–50 and >50 years of age), race, age at sexual debut (<16 versus ≥ 16 years of age), HAART duration (years on HAART), AIDS prior to inclusion, smoking status, number of lifetime sexual partners (<5 versus ≥ 5), use of hormonal contraceptives, and CD4 count at inclusion (<200, ≥200-349 and ≥350 cells/μL).

Predictors of ASCUS or worse (ASCUS+) were estimated by including the aforementioned variables, presence of cervical hrHPV and adherence to the general population CC screening program in the analysis. A subgroup analysis was performed in WLWH with ASCUS+ and cervical hrHPV only.

Duration of HAART, AIDS prior to inclusion and CD4 count are dependent covariates and where calculated using two models: A model where all variables but CD4 at inclusion was included and a model where duration of HAART and AIDS prior to inclusion were replaced by CD4. We only presented the OR of the CD4 count from the second model.

To account for multiple testing when comparing the distribution of genotypes, we applied Bonferoni correction. Therefore, in analyses of the 13 hrHPV genotypes *p*-values smaller than 0.05:13 (*p* < 0.0038) were considered statistically significant. In the remaining analyses *p*-values <0.05 were considered statistically significant. For category variables with more than two outcome categories (df > 1), we controlled for repeated testing by estimating the combined *p*-value. Individuals with missing explanatory values were excluded from the multiple regression analyses. The validity of the model was tested using the Hosmer and Lemeshow Goodness-of-Fit Test.

SAS statistical software version 9.3 (SAS Institute Inc., Cary, NC, USA) was used for data analysis. The matching of study participants and the HPV genotype distribution figures were performed in R 3.2.0 [32].

## Results

### Characteristics of the cohort

A total of 334 of the 1392 eligible WLWH in Denmark (in the DHCS) were included. At inclusion, median age and duration of HIV were 42.5 (IQR 36.8-48.3) and 11.3 (IQR 5.9-16.9) years (Table 1). Compared with WLWH from the DHCS not included, WLWH in SHADE were more likely to be sexually infected with HIV (*p* = 0.0015), have higher CD4 counts (*p* = 0.012), and increased probabilities of being on HAART with a suppressed viral load (*p* = 0.0042). Moreover, they had a higher uptake of both the annual HIV-and general population CC screening program (*p <* 0.0001 and *p <* 0.0001) and a higher probability of the latest cytology result being normal (*p <* 0.0001) (Table 1).

**Table 1:** Characteristics of included and not included women living with HIV (WLWH) in Denmark from the Danish HIV Cohort Study (DHCS)

	WLWH included in the study	WLWH not included in the study	*p*-value
Number of individuals	334 (24.0)	1,058 (76.0)	NA
Follow-up (years), median (IQR)	11.3 (5.9-16.9)	10.6 (5.5-15.9)	0.097
Follow-up time, total (person-years)	3,853	11,183	NA
Age at inclusion (years), median (IQR)	42.5 (36.8-48.3)	42.0 (35.5-48.2)	0.22
Race, n(%)			
White	141 (42.6)	398 (39.5)	0.041^a^
Asian	44 (13.3)	114 (11.3)	
Black	143 (43.2)	461 (45.7)	
Other	3 (0.9)	35 (3.5)	
(missing)	(3)	(50)	
Place of HIV transmission, *n*(%)			
Denmark	114 (37.8)	326 (34.8)	0.11
Europe + US	27 (8.9)	77 (8.2)	
Africa	128 (42.4)	416 (44.4)	
Asia	33 (10.9)	101 (10.8)	
Other	0 (0)	17 (1.8)	
(missing)	(32)	(121)	
Mode of transmission, *n*(%)			
Heterosexual	294 (91.6)	799 (83.6)	0.0015
IDU	16 (5.0)	106 (11.1)	
Other	11 (3.4)	51 (5.3)	
(missing)	(13)	(102)	
Age at sexual debut (years),			
mean (range)	17.3 (6–37)	-^b^	NA
Lifetime sexual partners, *n*(%)			
< 5	99 (29.6)	-^b^	NA
5–14	135 (40.4)		
15–25	45 (13.5)		
> 25	53 (15.9)		
Does not wish to respond	2 (0.6)		
(missing)	(0)		
CD4 count at inclusion (cells/μL), *n*(%)			
< 200	12 (3.9)	79 (9.1)	0.012
200–350	51 (16.5)	138 (15.9)	
> 350	247 (79.7)	650 (75.0)	
(missing)	(24)	(191)	
HAART at inclusion, *n*(%)			
Yes	317 (94.9)	866 (81.8)	<0.0001
No	17 (5.1)	192 (18.2)	
(missing)	(0)	(0)	
On HAART with			
HIV RNA < 40 copies/mL, *n*(%)			
Yes	250 (83.6)	576 (75.5)	0.0042
No	49 (16.4)	187 (24.5)	
(missing)	(18)	(103)	
Cervical cytology within the past 1 year, *n*(%)^c^			
Yes	124 (37.1)	225 (21.3)	<0.0001
No	210 (62.9)	833 (78.7)	
(missing)	(0)	(0)	
Cervical cytology within the past 3/5 years – depending on age for women age 23–65 years, *n*(%)^d^			
Yes	227 (67.7)	429 (40.6)	<0.0001
No	96 (28.7)	555 (52.5)	
Outside target age group	11 (3.3)	74 (7.0)	
(missing)	(0)	(0)	
Last cytology result, *n*(%)			
Normal	242 (72.5)	513 (48.5)	<0.0001
Abnormal	9 (2.7)	21 (2.0)	
No prior cytology obtained	83 (24.9)	524 (49.5)	
HPV vaccination prior to inclusion, *n*(%)			
Yes (4-valent HPV vaccine)	4 (1.2)	-^b^	NA
Yes (2-valent HPV vaccine)	0 (0)		
Yes (do not know name of vaccine)	1 (0.3)		
No	329 (98.5)		
(missing)	(0)		

*IDU* intravenous drug user, *NA* not applicable, *HAART* Highly active antiretroviral therapy, *HPV* Human papillomavirus

^a^There was no difference in distribution of race between groups if the category “other” was removed from the “Race” variable (*p* = 0.45), ^b^No information available, ^c^As recommended in women living with HIV (we studied the past year + a 3-month grace period), ^d^As recommended in the general population, where women aged 23–49 years were invited for cervical cancer screening every third year and women aged 50–65 years every fifth year (we studied the past 3/5 years + a 3-month grace period)

### HPV prevalence

**Fig. 1:**
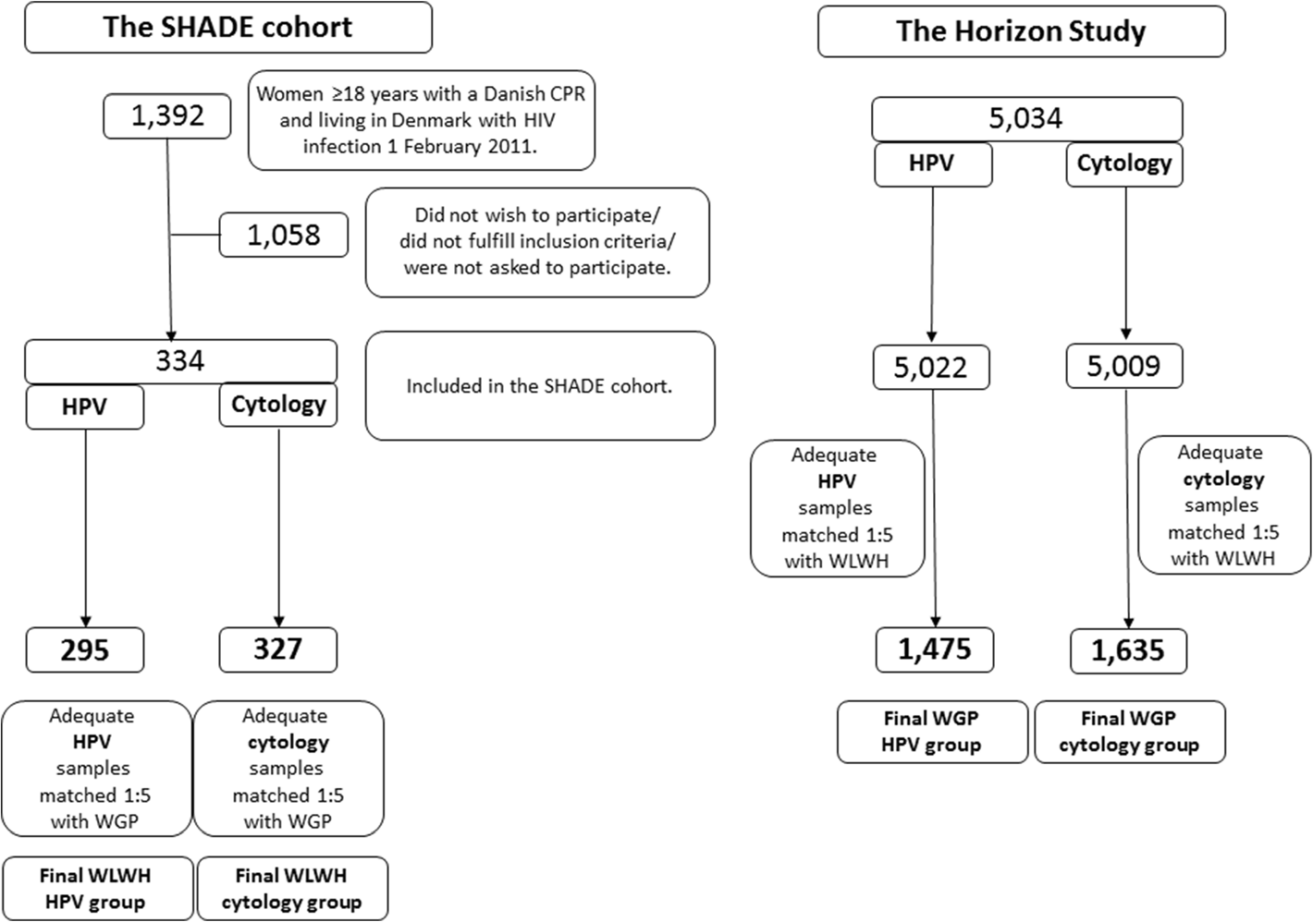
Flowchart of women living with HIV (WLWH) from the SHADE cohort and women from the general population (WGP) from the Horizon study matched 1:5 on prior screening history and age

Of 334 participants, 326 (97.6 %) had a cervical swab performed. Of these 295 (90.5 %) yielded sufficient DNA for analysis and were matched at 1:5 with 1475 WGP from the Horizon study (Fig. 1).

**Fig. 2:**
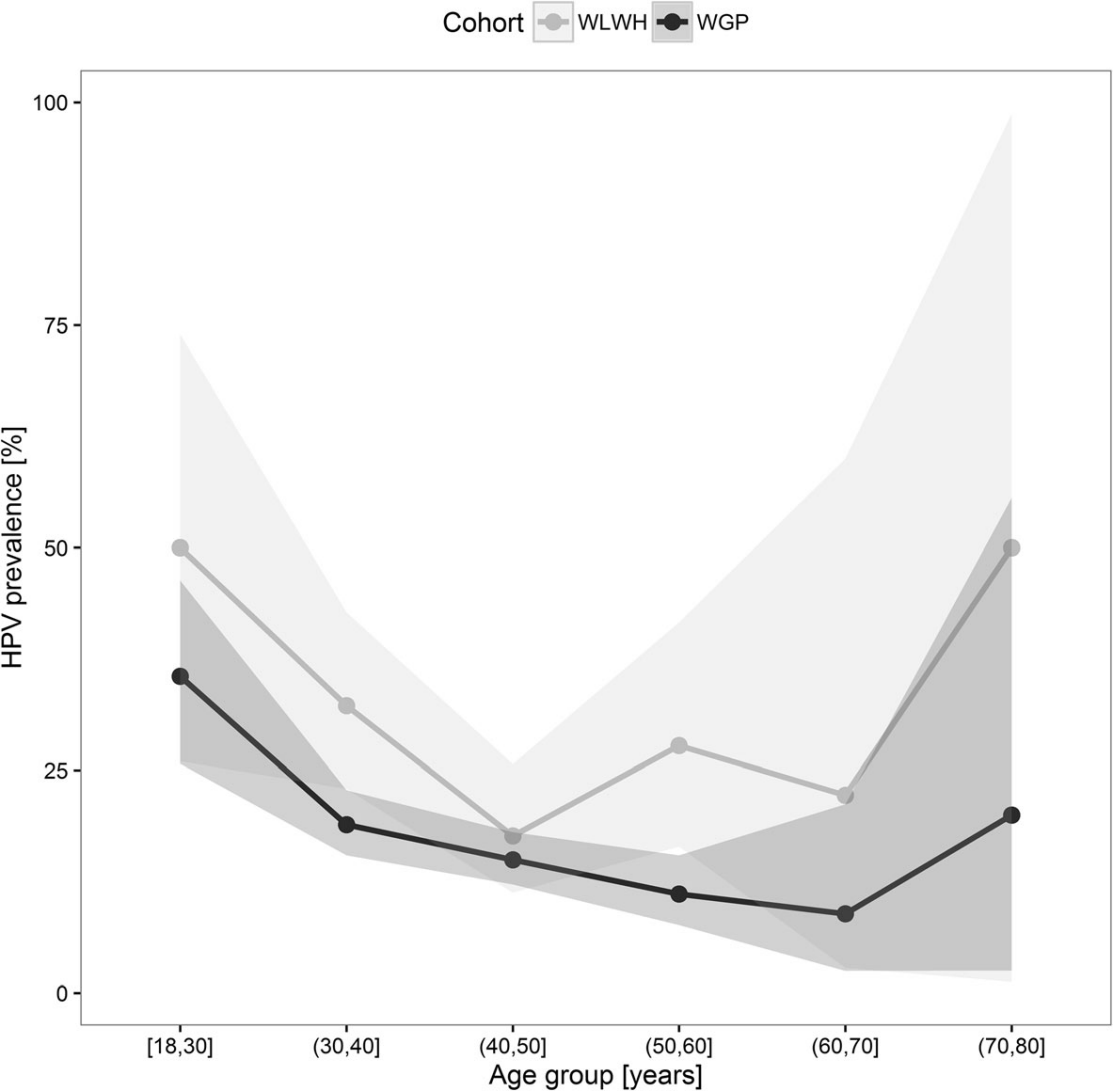
Prevalence of high-risk human papillomavirus (HPV) according to age group in women living with HIV (WLWH) and women from the general population (WGP)

Overall HPV prevalence was higher in WLWH versus WGP (26.4 % versus 16.6 %, *p* < 0.0001) (Table 2). Further, WLWH had a higher number of multiple infections with more genotypes diagnosed per sample (*p =* 0.030 and *p =* 0.047) (Table 2). Median age of WGP was due to the age matching criteria close to that of WLWH (stated above); 43.0 (IQR 37.0-49.0) years. The hrHPV prevalence according to age is shown in Fig. 2.

**Table 2:** Prevalence of high-risk human papillomavirus (HPV) in women with sufficient DNA for analyses in women living with HIV compared to women matched (1:5) on prior screening history and age from the general Danish population

	Women living with HIV 295	Women from the general population 1,475	*p*-value
High-risk HPV positive, *n*(%)			
Yes	78 (26.4)	245 (16.6)	<0.0001
No	217 (73.6)	1,230 (83.4)	
Number of genotypes, mean (range)	1.54 (1–4)	1.38 (1–5)	0.047
Number of infections, *n*(%)			
Single	48 (61.5)	182 (74.3)	0.030
Multiple (>1)	30 (38.5)	63 (25.7)	
All high-risk genotypes present targeted by the 4-valent HPV vaccine^a^, *n*(% of the HPV positive patients)			
Yes	8 (10.3)	52 (21.2)	0.030
No	70 (89.7)	193 (78.8)	
All high-risk genotypes present targeted by the 9-valent vaccine^b^, *n*(% of the HPV positive patients)			
Yes	42 (53.9)	155 (63.3)	0.14
No	36 (46.1)	90 (36.7)	
Presence of ≥1 high-risk genotypes targeted by the 4-valent vaccine^a^, *n*(% of the HPV positive patients)			
Yes	21 (26.9)	76 (31.0)	0.49
No	57 (73.1)	169 (69.0)	
Presence of ≥1 high-risk genotypes targeted by the 9-valent vaccine^b^, *n*(% of the HPV positive patients)			
Yes	61 (78.2)	194 (79.2)	0.85
No	17 (21.8)	51 (20.8)	

^a^Targeting HPV6, HPV11, HPV16 and HPV18. HPV6 and HPV11 are low-risk genotypes and not included in this analysis

^b^Targeting HPV6, HPV11, HPV16, HPV18, HPV31, HPV33, HPV45, HPV52, and HPV58. HPV6, and HPV11 are low-risk genotypes and not included in this analysis

More WGP would have been protected by the 4-valent HPV vaccine than WLWH (*p =* 0.030), whereas both groups of women would receive a similar degree of protection from the 9-valent HPV vaccine based upon their HPV genotype distribution (*p =* 0.14) (Table 2).

### HPV genotype distribution

**Fig. 3:**
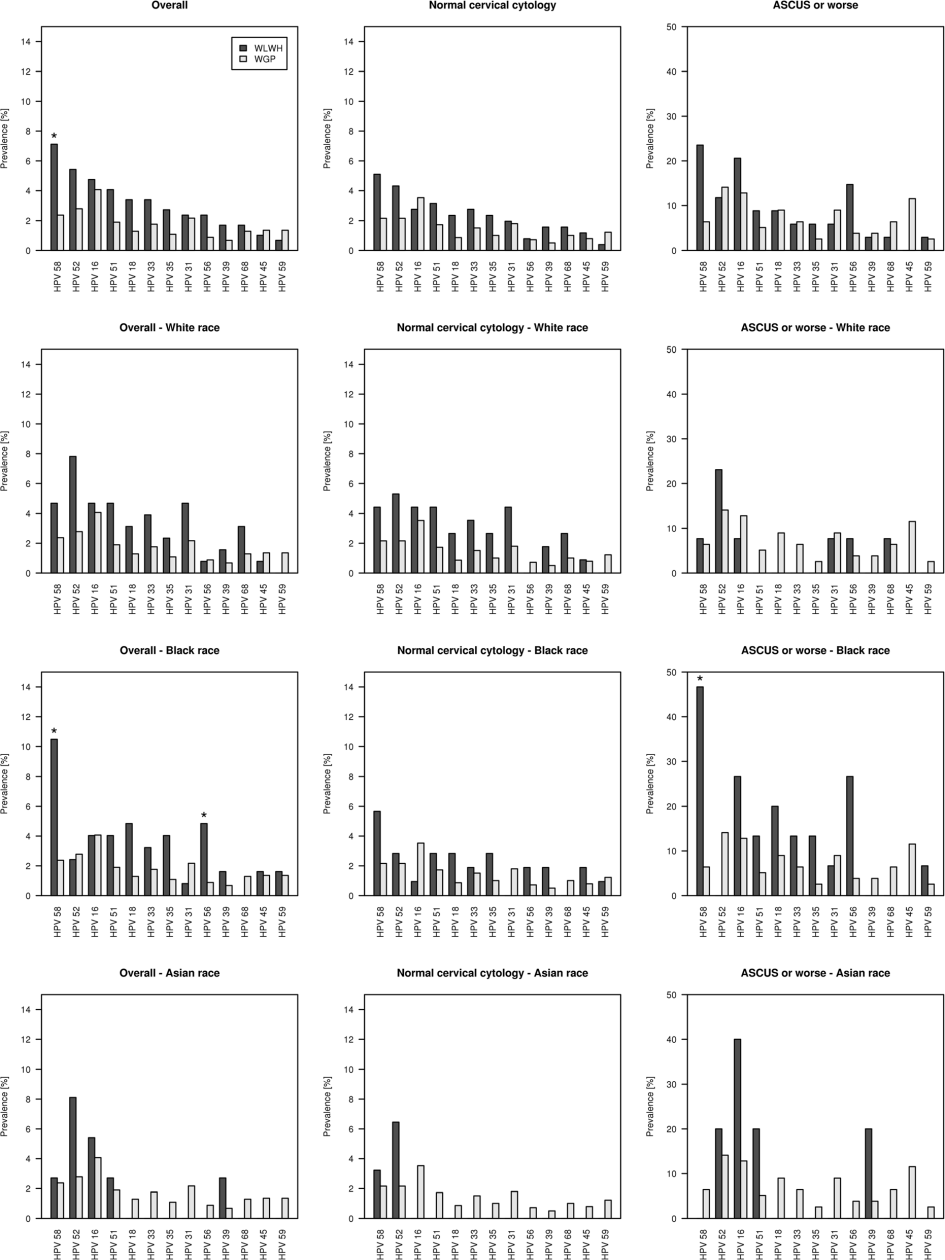
Cervical high-risk human papillomavirus (HPV) genotype distribution in women living with HIV (WLWH) compared to women from the general population (WGP); i) overall, ii) in women with normal cervical cytology, and iii) women with atypical cells of undetermined significance (ASCUS) or worse. Distribution is presented i) overall, ii) comparing WGP to WLWH of White race, iii) comparing WGP to WLWH of Black race and iv) comparing WGP to WLWH of Asian race. Please notice the different scale on the y-axis in the “ASCUS or worse” plots

Figure 3 shows the prevalence of hrHPV genotypes with respect to race: i) overall, ii) in WLWH and WGP with normal cytological findings, and iii) WLWH and WGP with ASCUS+. Overall, the six most frequent high-risk genotypes in WLWH were HPV58 (*n* = 21, 7.1 %), 52 (*n* = 16, 5.4 %), 16 (*n* = 14, 4.8 %), 51 (*n* = 12, 4.1 %), 18 (*n* = 10, 3.4 %) and 33 (*n* = 10, 3.4 %) versus 16 (*n* = 60, 4.1 %), 52 (*n* = 41, 2.8 %), 58 (*n* = 35, 2.4 %), 31 (*n* = 32, 2.2 %), 51 (*n* = 28, 1.9 %) and 33 (*n* = 26, 1.8) in WGP (Fig. 3, Additional file 1: Table S1).

### HPV genotype distribution according to race

There was no difference in prevalence of hrHPV in WLWH of White, Asian and Black race (26.6 %, 16.2 % and 29.0, (*p* = 0.30)). We compared genotype distribution in WLWH of different races and found that Black WLWH had a higher risk of HPV58 and 56 compared with WGP (*p* < 0.0001 and *p* = 0.0023) (Fig. 3), while no significant differences in hrHPV genotype distribution were found between White and Asian WLWH compared with WGP (Fig. 3).

### HPV genotype distribution in women with normal cytology

In women with normal cytology the hrHPV prevalence was 22.0 % in WLWH versus 13.0 % in WGP (*p* = 0.0004). HPV58 (*n* = 13, 5.1 %), 52 (*n* = 11, 4.3 %), 51 (*n* = 8, 3.1 %) versus HPV16 (*n* = 49, 3.5 %), 58 (*n* = 30, 2.2 %) and 52 (*n* = 30, 2.2 %) predominated in WLWH and WGP, respectively (Fig. 3, Additional file 2: Table S2).

### HPV genotype distribution in women presenting with ASCUS+

In WLWH and WGP with ASCUS+ 61.8 % versus 49.0 % were hrHPV-positive (*p* = 0.25).

Distribution of genotypes in descending order in WLWH and WGP presenting with ASCUS+ were HPV58 (*n* = 8, 23.5 %), 16 (*n* = 7, 20.6 %) and 56 (*n* = 5, 14.7 %) versus HPV52 (*n* = 11, 14.1 %), 16 (*n* = 10, 12.8 %) and 45 (*n* = 9, 11.5 %) (Fig. 3, Additional file 3: Table S3).

### Predictors of HPV

Short duration of HAART (adjusted OR per year 0.90 (95 % CI 0.84-0.96)), AIDS prior to inclusion (adjusted OR 3.61 (95 % CI 1.75-7.46)), ≥5 lifetime sexual partners (adjusted OR 2.20 (95 % CI 1.08-4.49)), sexual debut <16 years of age (adjusted OR 2.05 (95 % CI 1.03-4.10)) and CD4 < 350 cells/μL (adjusted OR 2.53 (95 % CI 1.20-5.40)) predicted prevalent hrHPV (Table 3).

**Table 3:** Unadjusted and adjusted odds ratios for predictors of cervical high-risk human papillomavirus (HPV) infection in women living with HIV with sufficient DNA for HPV analysis (*n* = 295)

Predictors of HPV^a^	HPV-positive group (*n* = 78)	HPV-negative group (*n* = 151)	Unadjusted odds ratios	*p*-value	Adjusted odds ratios^b^	*p*-value
Age at 1 February 2011 (inclusion), *n*(%)						
18-29 years	9 (11.5)	9 (4.2)	1.00	-	1.00	-
30-50 years	51 (65.4)	161 (74.2)	0.32 (0.12-0.84)	0.02	0.43 (0.14-1.33)	0.14
> 50 years	18 (23.1)	47 (21.7)	0.38 (0.13-1.12)	0.08	0.51 (0.13-1.92)	0.32
(missing)	0 (0)	(0)				
Combined p-value				0.068		0.32
Race, *n*(%)						
White	34 (44.7)	94 (44.1)	1.00	-	1.00	-
Asian	6 (7.9)	31 (14.6)	0.54 (0.21-1.40)	0.20	0.67 (0.23-1.92)	0.46
Black	36 (47.4)	88 (41.3)	1.13 (0.65-1.96)	0.66	0.38 (0.67-2.87)	0.38
(missing)	(2)	(4)	-	-		
Combined *p*-value				0.31		0.35
Sexual debut, *n*(%)						
≥ 16 years of age	51 (65.4)	160 (73.7)	1.00	-	1.00	-
< 16 years of age	27 (34.6)	57 (26.3)	1.49 (0.85-2.59)	0.16	2.05 (1.03-4.10)	0.042
(missing)	(0)	(0)	-			
HAART duration, (years)						
Median (IQR)	4.6 (2.0-10.4)	8.8 (4.6-12.2)	0.91 (0.86-0.96)	0.0013	0.90 (0.84-0.96)	0.0011
(missing)	(2)	(9)				
AIDS prior to inclusion, *n*(%)						
No	55 (71.4)	191 (88.4)	1.00	-	1.00	-
Yes	22 (28.6)	25 (11.6)	0.33 (0.17-0.63)	0.0007	3.61 (1.75-7.46)	0.0005
(missing)	(1)	(1)				
Smoking status, *n*(%)						
Current smoker/Ex-smoker	33 (42.3)	92 (42.4)	1.00	-	1.00	-
Never smoker	45 (57.7)	125 (57.6)	1.00 (0.59-1.69)	0.99	1.31 (0.65-2.63)	0.45
(missing)	(0)	(0)				
Number of lifetime sexual partners at inclusion, *n*(%)						
< 5	36 (25.0)	54 (36.0)	1.00	-	1.00	-
≥ 5	108 (75.0)	96 (64.0)	1.83 (1.00-3.36)	0.05	2.20 (1.08-4.49)	0.03
(missing)	(0)	(1)				
Use of hormonal contraceptives,						
*n*(%)	12 (8.3)	9 (6.0)	1.00	-	1.00	-
Yes	132 (91.7)	142 (94.0)	0.89 (0.33-2.38)	0.82	1.29 (0.40-4.10)	0.67
No	(0)	(0)				
(missing)						
CD4 count at inclusion (cells/μL),						
> 350	45 (69.2)	173 (83.2)	1.00	-	1.00	-
200-350	15 (23.1)	28 (13.5)	2.06 (1.02-4.18)	0.045	2.53 (1.20-5.40)	0.015
< 200	5 (7.7)	7 (3.4)	2.75 (0.83-9.06)	0.10	2.70 (0.78-9.33)	0.12
(missing)	(13)	(9)				
Combined *p*-value				0.0496		0.023

HAART = Highly active antiretroviral therapy

^a^Two models are shown in the table: Age, race, sexual debut, smoking status, number of lifetime sexual partners and use of hormonal contraceptives were included in both models, whereas HAART duration and AIDS prior to inclusion were included in the first model and replaced by CD4 at inclusion inthe second model, We only presented the ORs of the CD4 count from the second model, ^b^The validity of the model was tested using the Hosmer and Lemeshow Goodness-of-Fit Test

### Cervical cytological abnormalities

Five (1.5 %) of the 332 cytology samples received from WLWH were inadequate for evaluation leaving 327 for interpretation. These were matched with cytology results from 1635 WGP (Fig. 1). ASCUS+ was prevalent in 34 (10.4 %) versus 85 (5.2 %) WLWH and WGP, (*p* = 0.0003). Cytological abnormalities in WLWH and WGP were distributed as follows: ASCUS: 8 (2.5 %) versus 42 (2.6 %), (*p* = 0.90); LSIL: 20 (6.1 %) versus 23 (1.4 %), (*p* < 0.0001) and HSIL: 6 (1.8 %) versus 20 (1.2 %), (*p* = 0.38). No WLWH or WGP presented with carcinoma.

### Predictors of cytological abnormalities

HrHPV predicted ASCUS+ (adjusted OR 6.91 (95 % CI 2.91-16.42)) (Additional file 4: Table S4). However, in the subgroup of WLWH with hrHPV short duration of HAART predicted ASCUS+ (adjusted OR 0.83 (95 % CI 0.71-0.97)) (Additional file 5: Table S5).

In all adjusted analyses we checked the effect of missing values on outcome by adding an extra category with missing values. This had no impact on the estimates.

## Discussion

In this multicentre, cross-sectional cohort study of WLWH in Denmark, we found a higher prevalence of cervical hrHPV in WLWH compared with WGP matched on prior screening history and age. Further, WLWH had a higher number of hrHPV genotypes and more carried multiple hrHPV infections. Presence of ≥1 genotypes covered by the 9-valent HPV vaccine was higher in WLWH and a higher number of WGP had all present genotypes covered by the 4-valent HPV vaccine. WLWH had a different distribution of hrHPV genotypes and this difference was mainly attributed to WLWH of Black race. There was a higher risk of ASCUS+ in WLWH, due to a higher prevalence of LSIL. Finally, cervical HPV and ASCUS+ were predicted by short duration of HAART.

### HPV prevalence

Prevalent HPV infection is dependent on age with a peak prevalence in women in the early 20s followed by a steady decline and a second, but smaller peak in women ≥45 years of age attributed to either new acquisition or viral persistence [2, 14]. Therefore, we chose to match WLWH and WGP on age and prior cytology/histology results. The overall hrHPV prevalence in WLWH of 26.4 % was lower than the hrHPV prevalence found in the European MACH-1 collaborative group of WLWH with a prevalence close to 50 % [15], however participants in the MACH study were younger (median age: 35 versus 42.5 years), less likely to be on HAART (69.7 % versus 94.9 %) and a different assay (Hybrid Capture II) was used for HPV detection. On a global scale the highest prevalence of HPV in WGP with normal cytological findings is found in Africa (24 %) [14]. Though, the overall hrHPV prevalence was highest in WLWH of Black race, this difference was not significant.

### HPV genotype distribution

Studies of WGP across all continents have repeatedly identified hrHPV16 and 18 - accounting for about 70 % of all CCs [2, 6] – to be among the most prevalent [8]. In agreement with others we found HPV58 and 52 to be prevailing in WLWH [8, 16]. Though, the numbers were small, this higher risk of HPV58 was mainly carried by women of Black race, as no significant difference in genotype distribution, was detected when comparing WGP with WLWH of White and Asian race. While the 2-and 4-valent HPV vaccines are anticipated to reduce the burden of HPV-related cancers in WGP [13], the impact of these HPV vaccines in WLWH is less clear. More than 20 % of WGP had all genotypes present covered by the 4-valent HPV vaccine, whereas only one-tenth of genotypes were accounted for amongst WLWH. The novel 9-valent HPV-vaccine targeting HPV6, 11, 16, 18, 31, 33, 45, 52, and 58 has the potential to prevent about 90 % of CC cases in WGP if administered before sexual onset [33] might be better suited for the HIV population.

### Predictors of HPV

The molecular mechanisms leading to the increased risk of HPV in PLHIV are poorly understood [34]. Tugizov et al. suggested that HIV-proteins enable initial HPV infection by disrupting the epithelial tight junctions [34]. Moreover, immune defects associated with HIV infection probably contribute to the HPV pathogenesis by preventing spontaneous clearance of HPV [6]. A recent study found that in WLWH every month on HAART reduced the detection risk of any cervical HPV infection by 9 % [30]. Likewise, short duration of HAART predicted HPV in the current study. Moreover, sexual behavior such as early sexual debut and ≥5 lifetime sexual partners predicted prevalent HPV and so did variables associated with a compromised immune system such as CD4 < 350 cells/μL and AIDS prior to inclusion.

### Cytological abnormalities

Overall, WLWH had more cytological abnormalities, due to a higher prevalence of LSIL. A higher prevalence of LSIL, but not ASCUS and HSIL, in WLWH is also reported by others [35]. Most LSILs are transient and resolve within 1–2 years [36], and are most often not treated according to current Danish guidelines. A high proportion of WLWH included and not included in the study; 28.7 % and 52.5 %, had not been screened for CC in the preceding 3 to 5 years. We have previously assessed low screening attendance in this cohort of WLWH and support the idea of cytology as part of an annual medical HIV review, integration of HIV care and cervical screening in a single clinic setting and targeted public health messages aimed at health care professionals at HIV centres, general practitioners and WLWH [37]. However, the low screening attendance does not explain differences in cytological abnormalities between WLWH and WGP, since women were matched on prior screening history.

### Predictors of cervical dysplasia

Predictors of cervical dysplasia and CC are those also associated with HPV infection such as smoking, early sexual debut, number of lifetime sexual partners, hormonal contraceptives and STDs other than HPV [6, 7]. We have earlier reported that only a very few of WLWH in SHADE presented with STDs other than HPV [23] and therefore this variable was not adjusted for. Not surprisingly, hrHPV predicted ASCUS+. Since hrHPV causes most cases of dysplasia and basically all cases of CC [6], this variable could blur the effect of other covariates. We therefore performed a sensitivity analysis in WLWH with hrHPV, and in consistence with our findings regarding HPV we found that short duration of HAART predicted ASCUS+.

### Strengths and limitations

We have a very well-described cohort due to the DHCS and DPDB and were able to match on prior cytological outcomes. Compared to other Western countries Danish women represent a CC high-risk population [18], therefore comparison to WGP is essential in interpretation of results.

Possible limitations are that WLWH included in SHADE were more likely to comply with CC screening programs and to have a previous normal cytology result. Therefore estimates regarding cytological abnormalities in WLWH might be conservative. The effect of HAART duration can be confounded by other time-scales such as duration of HIV. Moreover, racial origin of the WGP group was not available for direct comparison to the SHADE cohort. Furthermore, a higher number of inadequate HPV samples were collected in the present study compared to ordinary CC screening samples included in the Horizon study, probably as a consequence of sampling by personnel less trained in gynecological routines. Finally, this is a cross-sectional study giving information on HPV infection at the time of one single sampling. The limitation in this design is that without previous or subsequent sampling results, any HPV infection observed may be a transient infection.

## Conclusions

WLWH had a higher risk of being cervical hrHPV positive, a higher frequency of multiple infections, a different genotype distribution and more cytological abnormalities than WGP. Cervical HPV and ASCUS+ were predicted by short duration of HAART.

## Additional files

Additional file 1: Table S1. Comparison of the overall prevalence of high-risk HPV genotypes in women living with HIV (WLWH) and age-matched women from the general population (WGP). (DOCX 20 kb)
Additional file 2: Table S2. Comparison of the prevalence of high-risk HPV genotypes in women living with HIV (WLWH) and women from the general population (WGP) *with normal cytological findings*. (DOCX 20 kb)
Additional file 3: Table S3. Comparison of the prevalence of high-risk HPV genotypes in women living with HIV (WLWH) and women from the general population (WGP) *with abnormal cytological findings (ASCUS or worse)*. (DOCX 20 kb)
Additional file 4: Table S4. Unadjusted and adjusted odds ratios for predictors of ASCUS or worse (ASCUS+) in women living with HIV with adequate cytology samples. (DOCX 27 kb)
Additional file 5: Table S5. Unadjusted and adjusted odds ratios for predictors of ASCUS or worse in women living with HIV with adequate cytology samples and positive for cervical high-risk human papillomavirus. (DOCX 26 kb)

## Abbreviations

CC: Cervical cancer
CI: Confidence interval
CRS: Civil Registration System
DF: Degrees of freedom
DHCS: Danish HIV Cohort Study
DPDB: The Danish Pathology Data Bank
HAART: Highly active antiretroviral therapy
HPV: Human papillomavirus
HVH: Hvidovre, Copenhagen University Hospital
MSM: Men who have sex with men
OR: Odds ratio
PIN: Personal identification number
PLHIV: People living with HIV
SHADE: **S**tudy on **H**IV, cervical **A**bnormalities and infections in women in **De**nmark
STD: Sexually transmitted diseases
WGP: Women from the general population
WLWH: Women living with HIV
