# Supplementary material for: Prevalence and distribution of cervical high-risk human papillomavirus and cytological abnormalities in women living with HIV in Denmark – the SHADE
Source: BMC Cancer. 2016 Nov 8;16:866. doi: 10.1186/s12885-016-2881-1 (PMC5100104; doi:10.1186/s12885-016-2881-1)
Supplement: Additional file 1: Table S1. — Comparison of the overall prevalence of high-risk HPV genotypes in women living with HIV (WLWH) and age-matched women from the general population (WGP). (DOCX 20 kb) [file 12885_2016_2881_MOESM1_ESM.docx]

Additional file 1 Table S1

Comparison of the overall prevalence of high-risk HPV genotypes in women living with HIV (WLWH) and age-matched women from the general population (WGP)

| High-risk Genotype | WLWH  n(%) | WGP  (%) | *p*-value |
| --- | --- | --- | --- |
| HPV 58 | 21 (7.1) | 35 (2.4) | 0.00014 |
| HPV 52 | 16 (5.4) | 41 (2.8) | 0.028 |
| HPV 16 | 14 (4.8) | 60 (4.1) | 0.63 |
| HPV 51 | 12 (4.1) | 28 (1.9) | 0.030 |
| HPV 18 | 10 (3.4) | 19 (1.3) | 0.019 |
| HPV 33 | 10 (3.4) | 26 ( 1.8) | 0.11 |
| HPV 35 | 8 (2.7) | 16 (1.1) | 0.047 |
| HPV 31 | 7 (2.4) | 32 (2.2) | 0.83 |
| HPV 56 | 7 (2.4) | 13 (0.9) | 0.036 |
| HPV 39 | 5 (1.7) | 10 (0.7) | 0.089 |
| HPV 68 | 5 (1.7) | 19 (1.3) | 0.58 |
| HPV 45 | 3 (1.0) | 20 (1.4) | 0.79 |
| HPV 59 | 2 (0.7) | 20 (1.4) | 0.56 |
