# Supplementary material for: Prevalence and distribution of cervical high-risk human papillomavirus and cytological abnormalities in women living with HIV in Denmark – the SHADE
Source: BMC Cancer. 2016 Nov 8;16:866. doi: 10.1186/s12885-016-2881-1 (PMC5100104; doi:10.1186/s12885-016-2881-1)
Supplement: Additional file 2: Table S2. — Comparison of the prevalence of high-risk HPV genotypes in women living with HIV (WLWH) and women from the general population (WGP) with normal cytological findings. (DOCX 20 kb) [file 12885_2016_2881_MOESM2_ESM.docx]

Additional file 2 Table S2

Comparison of the prevalence of high-risk HPV genotypes in women living with HIV (WLWH) and women from the general population (WGP)

*with normal cytological findings*

| High-risk Genotype | WLWH  (%) | WGP  (%) | *p*-value |
| --- | --- | --- | --- |
| HPV 58 | 13 (5.1) | 30 (2.2) | 0.016 |
| HPV 52 | 11 (4.3) | 30 (2.2) | 0.049 |
| HPV 16 | 7 (2.7) | 49 (3.5) | 0.71 |
| HPV 51 | 8 (3.1) | 24 (1.7) | 0.14 |
| HPV 18 | 6 (2.4) | 12 (0.9) | 0.047 |
| HPV 33 | 7 (2.7) | 21 (1.5) | 0.18 |
| HPV 35 | 6 (2.4) | 14 (1.0) | 0.11 |
| HPV 31 | 5 (2.0) | 25 (1.8) | 0.80 |
| HPV 56 | 2 (0.8) | 10 (0.7) | 1.00 |
| HPV 39 | 4 (1.6) | 7 (0.5) | 0.076 |
| HPV 68 | 4 (1.6) | 14 (1.0) | 0.51 |
| HPV 45 | 3 (1.2) | 11 (0.8) | 0.47 |
| HPV 59 | 1 (0.4) | 17 (1.2) | 0.34 |
