# Supplementary material for: Prevalence and distribution of cervical high-risk human papillomavirus and cytological abnormalities in women living with HIV in Denmark – the SHADE
Source: BMC Cancer. 2016 Nov 8;16:866. doi: 10.1186/s12885-016-2881-1 (PMC5100104; doi:10.1186/s12885-016-2881-1)
Supplement: Additional file 3: Table S3. — Comparison of the prevalence of high-risk HPV genotypes in women living with HIV (WLWH) and women from the general population (WGP) with abnormal cytological findings (ASCUS or worse). (DOCX 20 kb) [file 12885_2016_2881_MOESM3_ESM.docx]

Additional file 3 Table S3

Comparison of the prevalence of high-risk HPV genotypes in women living with HIV (WLWH) and women from the general population (WGP)

*with abnormal cytological findings (ASCUS or worse)^a^*

| High-risk Genotype | WLWH  (%) | WGP  (%) | *p*-value |
| --- | --- | --- | --- |
| HPV58 | 8 (23.5) | 5 (6.4) | 0.020 |
| HPV52 | 4 (11.7) | 11 (14.1) | 1.00 |
| HPV16 | 7 (20.6) | 10 (12.8) | 0.39 |
| HPV51 | 3 (8.8) | 4 (5.1) | 0.43 |
| HPV18 | 3 (8.8) | 7 (9.0) | 1.00 |
| HPV33 | 2 (5.9) | 5 (6.4) | 1.00 |
| HPV35 | 2 (5.9) | 2 (2.6) | 0.58 |
| HPV31 | 2 (5.9) | 7 (9.0) | 0.72 |
| HPV56 | 5 (14.7) | 3 (3.8) | 0.054 |
| HPV39 | 1 (2.9) | 3 (3.8) | 1.00 |
| HPV68 | 1 (2.9) | 5 (6.4) | 0.67 |
| HPV45 | 0 (0.0) | 9 (11.5) | 0.056 |
| HPV59 | 1 (2.9) | 2 (2.6) | 1.00 |

^a^ ASCUS = atypical cells of undetermined significance.
