# Supplementary material for: Prevalence and distribution of cervical high-risk human papillomavirus and cytological abnormalities in women living with HIV in Denmark – the SHADE
Source: BMC Cancer. 2016 Nov 8;16:866. doi: 10.1186/s12885-016-2881-1 (PMC5100104; doi:10.1186/s12885-016-2881-1)
Supplement: Additional file 4: Table S4. — Unadjusted and adjusted odds ratios for predictors of ASCUS or worse (ASCUS+) in women living with HIV with adequate cytology samples. (DOCX 27 kb) [file 12885_2016_2881_MOESM4_ESM.docx]

Additional file 4 Table S4

**Unadjusted and adjusted odds ratios for predictors of ASCUS or worse (ASCUS+) in women living with HIV with adequate cytology samples (n = 327).**

| Predictors of  **ASCUS+** | **ASCUS+**  **(n=34)** | **Normal cytology**  **(n=293)** | **Unadjusted**  **odds ratios** | ***p*-value** | **Adjusted**  **odds ratios^a,b^** | ***p*-value** |
| --- | --- | --- | --- | --- | --- | --- |
| **Age at 1 February 2011 (inclusion), (years), n(%)**  **18-29**  **30-49**  **≥50**  **(missing)**  **Combined p-value** | 4 (11.8)  25 (73.5)  5 (14.7)  (0) | 17 (5.8)  212 (72.4)  64 (21.8)  (0) | 1.00  0.50 (0.16-1.61)  0.33 (0.08-1.37) | -  0.25  0.13  0.31 | 1.00  1.10 (0.27-4.45)  0.77 (0.14-4.35) | -  0.89  0.76  0.83 |
| **Race, n(%)**  **White**  **Asian**  Black  (missing)  **Combined p-value** | 13 (39.4)  5 (15.1)  15 (45.5)  (1) | 126 (43.8)  38 (13.2)  124 (43.0)  (5) | 1.00  1.28 (0.43-3.81)  1.17 (0.54-2.57) | -  0.66  0.69  0.88 | 1.00  1.51 (0.42-5.41)  1.04 (0.37-2.91) | -  0.52  0.94  0.80 |
| Sexual debut, n(%)  **< 16 years of age**  **≥ 16 years of age**  **(missing)** | 9 (26.5)  25 (73.5)  (0) | 80 (27.3)  213 (72.7)  (0) | 1.00  1.04 (0.47-2.33) | -  0.92 | 1.00  1.21 (0.44-3.31) | -  0.71 |
| HAART duration, (years)  **Median (IQR)** | 3.59 (1.07-10.73) | 8.07 (3.96-11.93) | 0.90 (0.83-0.97) | **0.0092** | 0.94 (0.85-1.03) | 0.16 |
| **AIDS prior to inclusion, n(%)**  **No**  **Yes**  **(missing)** | 26 (76.5)  8 (23.5)  (0) | 250 (85.9)  41 (14.1)  (2) | 1.00  0.53 (0.23-1.26) | -  0.15 | 1.00  1.38 (0.52-3.69) | -  0.52 |
| **Smoking status, n(%)**  Current smoker/ Ex-smoker  Never smoker  **(missing)** | 15 (44.1)  19 (55.9)  (0) | 121 (41.3)  172 (58.7)  (0) | 1.00  0.89 (0.44-1.82) | -  0.75 | 1.00  0.57 (0.22-1.48) | -  0.25 |
| **Number of lifetime sexual partners at inclusion, n(%)**  **<5**  **≥5**  **(missing)** | 13 (38.2)  21 (61.8)  (0) | 83 (28.5)  208 (71.5)  (2) | 1.00  0.65 (0.31-1.35) | -  0.24 | 1.00  0.52 (0.20-1.31) | -  0.17 |
| **Use of hormonal contraceptives, n(%)**  **Yes**  **No**  **(missing)** | 3 (8.8)  31 (91.2) | 18 (6.1)  275 (93.9) | 1.00  0.68 (0.19-2.43) | -  0.55 | 1.00  0.62 (0.14-2.68) | -  0.52 |
| **High-risk HPV, n(%)**  **No**  **Yes**  **(missing)** | 13 (38.2)  21 (61.8)  (0) | 233 (80.6)  56 (19.4)  (4) | 1.00  6.72 (3.17-14.24) | -  **<0.0001** | 1.00  6.91 (2.91-16.42) | **-**  **<0.0001** |
| **Adherence to general population cervical cancer screening program, n(%)^c^**  **Yes**  **No**  **(missing)** | 19 (55.9)  15 (44.1)  (0) | 205 (70.0)  88 (30.0)  (0) | 1.00  1.84 (0.89-3.78) | -  0.06 | 1.00  1.33 (0.56-3.17) | **-**  0.51 |
| **CD4 count at inclusion (cells/μL), n(%)**  **>350**  **200-350**  **<200**  **Missing**  **Combined p-value** | 18 (69.2)  6 (23.1)  2 (7.7)  (8) | 225 (81.0)  44 (15.8)  9 (3.2)  (15) | 1.00  1.71 (0.64-4.54)  2.78 (0.56-13.84) | -  0.29  0.21  0.31 | 1.00  1.51 (0.52-4.41)  3.02 (0.53-17.31) | -  0.45  0.22  0.40 |

**ASCUS=**[**atypical cells of undetermined significance**](http://womenshealth.about.com/cs/papsmears/g/defascus.htm)**, HPV=Human papillomavirus, HAART=Highly active antiretroviral therapy.**

**^a^ Two models are shown in the table: Age, race, mode of transmission, smoking status, number of lifetime sexual partners and use of hormonal contraceptives were included in both models, whereas HAART duration and AIDS prior to inclusion were included in the first model and replaced by CD4 at inclusion in the second model. We only presented the ORs of the CD4 count from the second model, ^b^ The validity of the model was tested using the Hosmer and Lemeshow Goodness-of-Fit Test, ^c^ Women <23 and >64 years of age are not within the target age group of the general population cervical cancer screening program and are therefore placed in the “Yes” category.**
